# The association between obesity and telomere shortening is mediated through total bilirubin

**DOI:** 10.1186/s40842-026-00307-2

**Published:** 2026-07-10

**Authors:** Bin Zhou, Lei Ding, Xuerong Sun, Wei Hua, Daoliang Zhang, Zhiyong Liao

**Affiliations:** 1 Department of Cardiology, Fuwai Shenzhen Hospital, Chinese Academy of Medical Sciences, Shenzhen, Guangdong Province 518057 China; 2 Fuwai Hospital, National Centre for Cardiovascular Diseases, National Clinical Research Center of Cardiovascular Diseases, State Key Laboratory of Cardiovascular Disease, Chinese Academy of Medical Sciences and Peking Union Medical College, Beijing, 100037 China; 3 Department of Cardiology, Beijing Chaoyang Hospital, Capital Medical University, Beijing, 100020 China

**Keywords:** Leukocyte telomere length, Body mass index, Total bilirubin, Oxidative stress, Mediation analysis

## Abstract

**Background:**

Both obesity and oxidative stress are closely linked to leukocyte telomere length (LTL) shortening, yet the specific mediating role of total bilirubin in this association remains insufficiently explored. This study aimed to quantify the extent to which total bilirubin, a potent endogenous antioxidant, mediates the association between obesity and LTL.

**Methods:**

We analyzed data from 6,717 adults participating in the National Health and Nutrition Examination Survey 1999–2002. Causal mediation analysis with 1,000 bootstrap iterations was employed to investigate the mediating effect of serum total bilirubin on the association between body mass index (BMI) and LTL.

**Results:**

In the fully adjusted model, BMI was significantly and inversely associated with LTL, with a total effect of -3.532 base pairs (bp) per 1-unit increase in BMI (95% confidence interval [CI]: -6.225 to -1.178; *P* = 0.002). Serum total bilirubin significantly mediated this association (average causal mediation effect = -0.446 bp; 95% CI: -0.726 to -0.237; *P* < 0.001). The estimated proportion of the association explained by bilirubin was 13.2% (95% CI: 5.5% to 41.2%; *P* = 0.002). A series of sensitivity analyses confirmed the robustness and stability of these mediation findings.

**Conclusions:**

Total bilirubin serves as a significant statistical mediator in the relationship between obesity and LTL shortening. However, given the cross-sectional design of this study, further longitudinal investigations are required to confirm these associations and establish causality.

**Supplementary Information:**

The online version contains supplementary material available at 10.1186/s40842-026-00307-2.

## Introduction

Obesity is a global public health problem with a growing prevalence [1]. Numerous comorbidities, including cardiovascular disease, type 2 diabetes, several common cancers, and sleep apnea/sleep-disordered breathing, have been linked to obesity [2]. Obesity is linked to a greater risk of morbidity and mortality, as well as a shorter lifespan [2]. In health screenings and epidemiological surveys, body mass index (BMI) is an easily accessible and commonly used indicator of obesity [3]. Previous studies have reported the inverse association of BMI with leukocyte telomere length (LTL) [4–6]. However, the underlying mechanisms remain unknown.

Telomeres are nucleoprotein structures at the ends of chromosomes that stabilize the chromosome and protect it from end-degrading enzymes. They become progressively shortened with cell division, leading to a decrease in telomere length with age [7]. As a result, LTL has been used as a marker of biological aging, particularly cardiovascular aging [8–10].

Furthermore, increasing evidence has shown that obesity is associated with increased oxidative stress and decreased antioxidant capacity [11, 12]. Oxidative stress refers to an imbalance between the generation of reactive oxygen species and their elimination by the antioxidant system [13]. Oxidative stress is also linked to the shortening of telomeres and is involved in cell aging [14]. Bilirubin, which is one of the most potent endogenous antioxidants, is the main end product of heme catabolism in the intravascular compartment [15]. Low levels of bilirubin suggest a prooxidant state due to depleted antioxidant defenses. Thus, a decreased bilirubin concentration exacerbates oxidative stress-related diseases, including metabolic syndrome [16] and cardiovascular diseases [17]. In addition, a decreased bilirubin concentration was reported to be associated with LTL shortening [18].

Despite the complex link between obesity, LTL, and oxidative stress, no studies have reported a potential mediating role of oxidative stress in the BMI‒LTL association. In the present study, we aimed to explore whether total bilirubin mediates the association between obesity and telomere shortening using a large sample of randomly selected US adults who participated in the National Health and Nutrition Examination Survey (NHANES) from 1999 to 2002 to elucidate whether oxidative stress is potentially a medicating role linking obesity to LTL attrition.

## Materials and methods

### Study design and population

The NHANES is a major program of the National Center for Health Statistics (NCHS), Centers for Disease Control and Prevention (CDC), which focuses on the health and nutritional status of a nationally representative sample of the U.S. civilian population. Since 1999, the continuous NHANES program has been conducted with multistage cross-sectional surveys in two-year cycles that include information about the interviews (demographic, socioeconomic, dietary, and health-related questions), physical examinations (medical, dental, and physiological measurements), and laboratory tests administered by highly trained medical personnel. Each NHANES participant provided written informed consent. The NCHS Ethics Review Board approved the collection of the NHANES data and the public release of the files. Descriptions of the study design, protocols, and survey methods of the NHANES are available in detail on the website (https://www.cdc.gov/nchs/nhanes/index.htm).

**Fig. 1:**
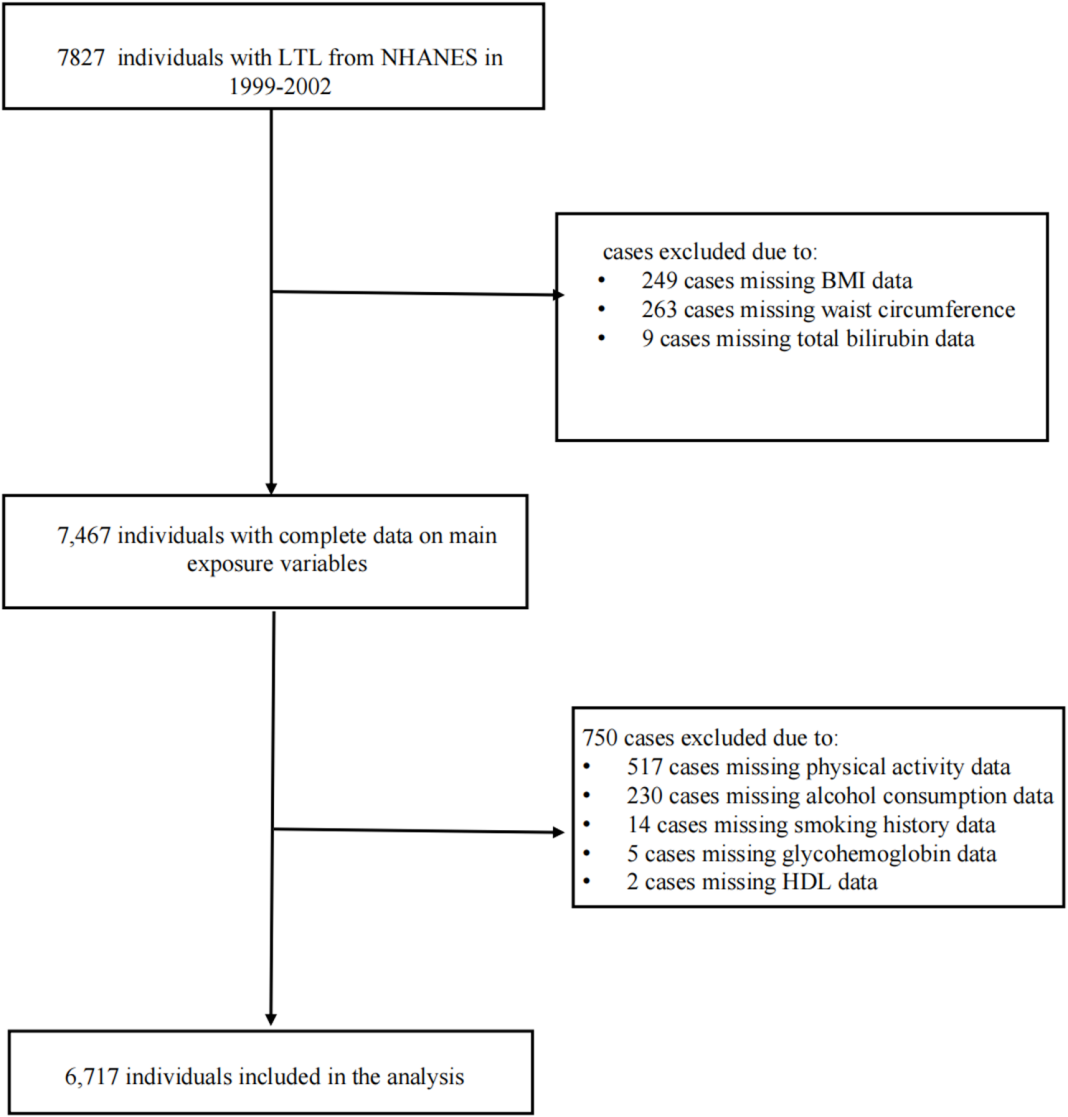
Flowchart of the study population

The NHANES participants from 1999 to 2002 were enrolled into the present study cohort if the data on BMI, waist circumference, total bilirubin, and LTL were complete and if the data on any of the covariates (age, sex, race, cigarette smoking status, alcohol consumption, physical activity, alanine aminotransferase [ALT], aspartate aminotransferase [AST], C-reaction protein [CRP], high-density lipoprotein cholesterol [HDL], and glycohemoglobin) were available. The initial pool comprised 21,004 surveyed individuals. Among the 21,004 participants initially enrolled in NHANES 1999–2002, we first identified 7,827 individuals with available LTL measurements. From this genetic subsample, we further excluded participants with missing data on primary exposure and mediator variables: BMI (*n* = 249), waist circumference (*n* = 263), and total bilirubin (*n* = 9), leaving 7,467 individuals. This step yielded 7467 individuals with complete data on the main exposure (BMI, waist circumference), mediator (total bilirubin), and outcome (LTL). From the cohort of 7467, a further 750 individuals were excluded due to missing data on important covariates: physical activity data (*n* = 517), alcohol consumption data (*n* = 230), smoking data (*n* = 14), glycohemoglobin data (*n* = 5), and HDL data (*n* = 2) (Fig. 1). The final analytical sample comprised 6,717 participants. This was derived by applying strict listwise deletion to the total available LTL dataset, excluding individuals with missing data on primary study variables (obesity and serum bilirubin) or any of the adjusted covariates. This complete-case approach was adopted to ensure the integrity and consistency of the mediation models.

### Main exposure and measurement

BMI was calculated as weight (kg) divided by the square of the patient’s height (m^2^) and expressed as kg/m^2^. For sensitivity analysis, waist circumference (in centimeters) was incorporated as an alternative indicator of central adiposity. Waist circumference was measured by trained NHANES personnel using a standardized protocol: a tape measure was placed horizontally at the uppermost lateral border of the iliac crest (hip bone) during minimal respiration, ensuring accuracy and reproducibility.

The NCHS supplied aliquots of purified DNA for this study. DNA extraction from whole blood was performed following the Puregene (D-50 K) kit protocol (Gentra Systems, Inc., Minneapolis, Minnesota), and the extracted DNA was subsequently stored at -80 °C. Mean LTL was assessed via quantitative polymerase chain reaction (qPCR) by calculating the relative telomere repeat copy number to single-copy gene copy number (T/S ratio); comprehensive methodological details for this established procedure are available in prior publications [19, 20]. Each DNA sample underwent triplicate qPCR assays conducted on three separate days. Measurements were performed in duplicate wells per assay, yielding a total of six data points per sample. Assay plates were processed in batches of three, with a study design ensuring that no two plates were grouped together more than a single time. Every plate included 96 control wells, and any run with eight or more invalid controls was deemed a failure and excluded from subsequent analysis (affecting less than 1% of runs). The T/S ratio for each sample was averaged from the replicate measurements. Potential outliers were identified as the highest and lowest values within the dataset. A new mean was computed after excluding these potential outliers. A value was formally classified as an outlier if the absolute logarithm of the ratio between the recalculated mean and the potential outlier’s value exceeded 0.4. The vast majority of samples (98.7%) contained no outliers. The laboratory performing the LTL measurements was blinded to all sample characteristics. The inter-assay coefficient of variation for the LTL measurements was 4%. The T/S ratio was converted to kilobase pairs using the formula: 3,274 + 2,413 * (T/S) / 1,000 [21]. To enhance biological interpretability, relative LTL (T/S ratio) was converted into absolute LTL in base pairs (bp) using the formula. Consequently, all regression coefficients and mediation effects are reported in bp. It is important to note that this conversion factor is specific to the laboratory and assay conditions; therefore, the reported kilobase values should be regarded as approximations. While this does not affect the internal validity of the study’s findings, the absolute telomere length values are not directly comparable to those reported in other studies that used different calibration methods. All assays were carried out in the Blackburn Laboratory at the University of California, San Francisco.

To quantify total bilirubin levels, bilirubin is coupled with a diazonium salt in a strongly acidic medium (pH 1 to 2). The intensity of the color of the azobilirubin produced is proportional to the total bilirubin concentration and can be measured photometrically. The Beckman Synchron LX20 uses a timed-endpoint Diazo method to measure the concentration of total bilirubin in serum or plasma. In the reaction, bilirubin reacts with the diazo reagent in the presence of caffeine, benzoate, and acetate as accelerators to form azobilirubin. The system monitors the change in absorbance at 520 nm at a fixed time interval. This change in absorbance is directly proportional to the concentration of total bilirubin in the sample. The total bilirubin measurement method is available at the following website: https://wwwn.cdc.gov/Nchs/Nhanes/2001-2002/L40_2_B.htm.

### Covariates

The considered variables, including demographic information, lifestyle factors, and some biochemical indices, were reported as important factors in previous studies [22–28] and extracted as covariates in the present study. Data on demographics, cigarette smoking status, alcohol consumption, and physical activity were collected with the questionnaire interviews, and data on ALT, AST, CRP, HDL, and glycohemoglobin levels were collected with the laboratory tests. The variable codebooks of the surveys are available on the website (http://www.cdc.gov/nchs/nhanes.htm).

### Statistical methods

The CDC recommendations were followed for all the statistical analyses (https://wwwn.cdc.gov/nchs/nhanes/tutorials/default.aspx). Each person who took part in the NHANES was given a sample weight [29]. As a result, we employed the proposed weighting methodology to account for significant variance. The data are displayed as the means ± standard deviations or proportions. To find differences between age groups, we employed either a weighted chi-square test (categorical variables) or a weighted linear regression model (continuous variables).

A multiple linear regression model was used to assess the associations among BMI, total bilirubin, and LTL. The selection of covariates was guided by principles of causal inference for mediation analysis to obtain an unbiased estimate of the natural indirect effect. Our primary aim was to adjust for true confounders while avoiding the adjustment of mediators or colliders, which can introduce significant bias. The following variables were included in all models and are not on the causal pathway: age, sex, race, cigarette smoking status, alcohol consumption, physical activity. Model 1 was conducted to assess the effects of BMI on LTL, adjusting for age, sex, race, cigarette smoking status, alcohol consumption, physical activity, ALT, AST. Model 2 was used to evaluate the effects of BMI and total bilirubin on LTL, adjusting for the variables in Model 1 plus total bilirubin. Model 3 was performed to assess the effects of BMI on bilirubin, adjusting for the variables in Model 1. The 95% confidence intervals (CI) were calculated to show the impact at each level.

A causal mediation analysis was performed to estimate the potential mediating role of serum bilirubin in the association between BMI and LTL using a counterfactual framework. This method allows for a more robust decomposition of the total effect into the average causal mediation effect (ACME) and the average direct effect (ADE), even in the presence of complex confounding. The mediation analysis was implemented using the mediation package in R. We utilized a non-parametric bootstrap procedure with 1,000 iterations to derive the point estimates and 95% CI for all effects. The proportion mediated was calculated as the ratio of ACME to the total effect, representing the extent to which serum bilirubin statistically explains the obesity-LTL association. The model was fully adjusted for age, sex, race/ethnicity, cigarette smoking, alcohol consumption, physical activity, glycohemoglobin, CRP, HDL, and liver enzymes (ALT and AST).

### Sensitivity analysis

To ensure the robustness of our primary findings and minimize potential biases, a series of sensitivity analyses were performed. First, selection bias was evaluated by comparing the sociodemographic and clinical characteristics of the included participants with the excluded eligible population to ensure the representativeness of our final analytical sample. Second, considering that previous literature has reported potential non-linear associations between serum bilirubin and LTL [18], we utilized restricted cubic splines (RCS) to characterize the dose-response relationship. To further ensure that the observed mediation effect was not disproportionately driven by individuals with exceptionally low bilirubin levels, an additional sensitivity analysis was conducted by excluding the lowest 10% of the bilirubin distribution. Third, to exclude the influence of pathological hyperbilirubinemia or genetic conditions such as Gilbert’s syndrome, the analyses were repeated after excluding individuals with total bilirubin levels exceeding 1.2 mg/dL. Fourth, incremental adjustment models were constructed to evaluate the stability of the mediation proportion across various metabolic and inflammatory backgrounds. Fifth, to distinguish the antioxidant consumption pathway from obesity-related hepatic dysfunction (e.g., Non-alcoholic fatty liver disease [NAFLD]), we performed a sensitivity analysis restricted to participants with normal liver enzyme levels. Finally, waist circumference was utilized as an alternative exposure metric to verify the generalizability of the identified Obesity-Bilirubin-LTL axis regardless of the adiposity definition used.

Although we utilized a formal mediation analysis framework, given the cross-sectional nature of the NHANES 1999–2002 data, the results should be interpreted as statistical associations rather than definitive causal pathways. The term ‘mediation’ is used to describe the statistical partitioning of the total association between obesity and LTL. The key assumptions required for a causal interpretation include the condition of no unmeasured confounding for all relevant pathways. For the telomere length assay in the 1999–2002 cycles, no subsample-specific weight was provided by NCHS. Therefore, in line with prior analyses of this dataset, the Mobile Examination Center (MEC) examination weights were applied to ensure national representativeness. All analyses accounted for the complex survey design of NHANES. Sampling weights, stratification, and clustering were incorporated to obtain nationally representative estimates and appropriate standard errors using R survey package. All the analyses were performed using R version 4.0.3 (The R Foundation for Statistical Computing, Vienna, Austria). A P value < 0.05 was considered significant, and all tests were two-sided.

## Results

### Baseline characteristics

Table 1 shows the weighted baseline characteristics of the study participants. The mean age of the enrolled individuals was 44.9 years, 52.8% were male adults, and 72.9% were non-Hispanic white. The reduction in sample size from 7827 to 6,717 was primarily due to missing values across exposure, mediator, and covariate variables. While some sociodemographic differences existed (e.g., age and physical activity), the two groups remained comparable in key metabolic indicators such as BMI and telomere length (Supplementary Table S1).

**Table 1:** Weighted baseline characteristics

Variables	Overall
^a^ N	6717
Age, years (mean ± sd)	44.9 ± 16.4
Sex, male (%)	52.8
Race/ethnicity (%)	
Non-Hispanic white	72.9
Non-Hispanic black	9.1
Mexican American	6.9
Other Hispanic	7.2
Other	4.0
Cigarette smoking (%)	
Never	50.5
Past	25.0
Current	24.5
Alcohol consumption, gm (mean ± sd)	11.2 ± 36.4
^b^ Physical activity, MET-based rank (%)	
Sedentary	19.6
Low	28.0
Moderate	19.4
High	33.0
BMI, kg/m^2^ (mean ± sd)	28.1 ± 6.3
Waist circumference, cm (mean ± sd)	95.9 ± 15.7
LTL, base pairs (mean ± sd)	5834.3 ± 665.5
Total bilirubin, mg/dL (mean ± sd)	0.7 ± 0.3
ALT, U/L(mean ± sd)	26.3 ± 34.0
AST, U/L(mean ± sd)	24.5 ± 17.5
CRP, mg/dL (mean ± sd)	0.4 ± 0.7
HDL, mg/dL (mean ± sd)	50.9 ± 15.3
Glycohemoglobin, % (mean ± sd)	5.4 ± 0.9

Note:

Mean ± SD (standard deviation) for continuous variables, P value was calculated by weighted linear regression model. Number (%) for categorical variables, P value was calculated by weighted chi-square test

^a^ Unweighted number of observations in dataset

^b^ Physical activity categories were based on the distribution of MET -minute levels for the present NHANES sample

Abbreviations: BMI, body mass index; ALT, alanine aminotransferase; AST, aspartate aminotransferase; CRP, C-reaction Protein; HDL, high-density lipoprotein cholesterol; LTL: Leukocyte telomere length

### Associations among BMI, total bilirubin, and LTL

The results of the multiple linear regression analysis are presented in Table 2. In Model 1, BMI was significantly and inversely associated with LTL. Each 1-unit increase in BMI corresponded to a total LTL shortening of 3.532 bp (beta = -3.532, 95% CI: -5.965 to -1.098, *P* = 0.0045). In Model 2, after additional adjustment for the mediator (total bilirubin), the direct association between BMI and LTL remained statistically significant (beta = -3.066, 95% CI: -5.506 to -0.625, *P* = 0.0138). Simultaneously, total bilirubin was found to be significantly and positively associated with LTL (beta = 119.069 bp per 1 mg/dL increase in bilirubin, 95% CI: 66.682 to 171.456, *P* < 0.001). Furthermore, Model 3 demonstrated that increased BMI was significantly correlated with a decrease in serum total bilirubin levels (beta = -0.004 mg/dL, 95% CI: -0.005 to -0.003, *P* < 0.001).

**Table 2:** Multiple linear regression analysis

	Total bilirubin (M, mediator)		LTL (Y, outcome variable)	
	β (95% CI)	*P* value	β (95% CI)	*P* value
**Model 1**				
BMI (X, independent variable)	-	-	-3.532 (-5.965~-1.098)	0.0045
**Model 2**				
BMI (X, independent variable)	-	-	-3.066 (-5.506~-0.625)	0.0138
Total bilirubin (M, mediator)	-	-	119.069 (66.682 ~ 171.456)	< 0.001
**Model 3**				
BMI (X, independent variable)	-0.004 (-0.005~-0.003)	< 0.001	-	-

Note: Model 1: adjusted for for age, sex, race, cigarette smoking, alcohol consumption, physical activity, ALT, AST. Model 2: adjusted variables in model 1 plus total bilirubin. Model 3: adjusted variables in model 1. Abbreviations are shown in Table 1

### The mediating effect of total bilirubin on the association between BMI and LTL

**Fig. 2:**
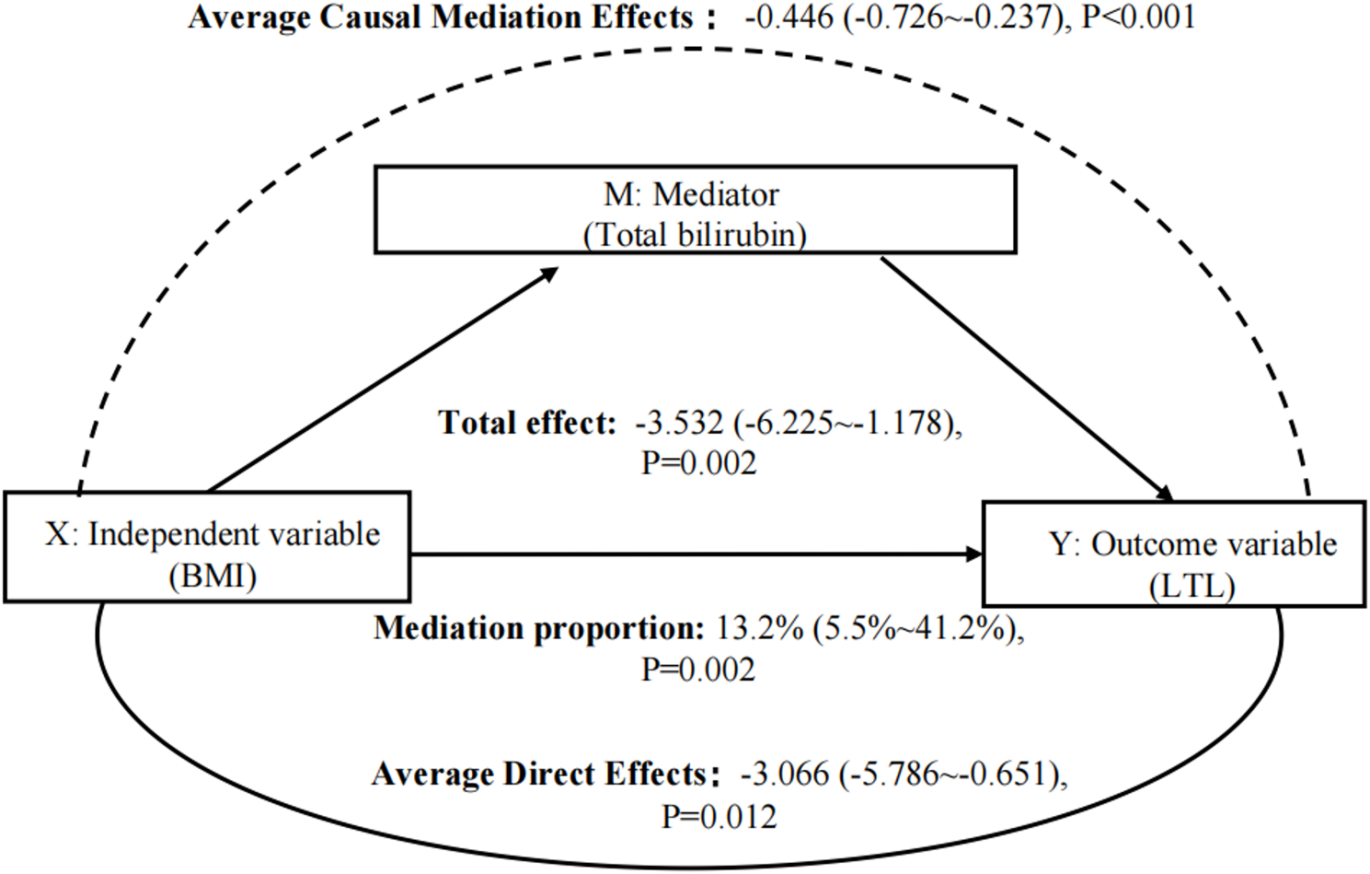
Mediation analysis of the association between BMI and LTL mediated by serum total bilirubin. The model was fully adjusted for age, sex, race, cigarette smoking, alcohol consumption, physical activity, ALT, AST, CRP, HDL, and glycohemoglobin. Abbreviations as Table 1

The results of the formal mediation analysis, which utilized a non-parametric bootstrap approach with 1,000 repetitions, are illustrated in Fig. 2. The total effect of BMI on LTL was − 3.532 bp (95% CI: -6.225 to -1.178, *P* = 0.002). The ACME, representing the indirect pathway through serum total bilirubin, was significantly estimated at -0.446 bp (95% CI: -0.726 to -0.237, *P* < 0.001). The ADE of BMI on LTL was − 3.066 bp (95% CI: -5.786 to -0.651, *P* = 0.012). Collectively, serum total bilirubin statistically accounted for 13.2% of the total association between obesity and telomere shortening (95% CI: 5.5% to 41.2%, *P* = 0.002). To facilitate biological interpretation, the mediation effects were translated into base pairs (bp). A 5-unit increment in BMI was associated with a total LTL reduction of 17.66 bp (95% CI: 5.89 to 31.13 bp). The indirect effect mediated by serum bilirubin accounted for a loss of 2.33 bp (95% CI: 1.18 to 3.63 bp), representing 13.2% of the total association (*P* < 0.001).

### Sensitivity analysis

First, to address the potential non-linearity of the mediator, we performed a RCS analysis, which identified a threshold saturation effect at 0.582 mg/dL (Supplementary Fig. S1). Below this level, a steep positive association between bilirubin and LTL was observed, while the relationship reached a plateau at higher concentrations (Supplementary table S2). Furthermore, to ensure the observed effect was not solely driven by extreme bilirubin deficiency, we conducted a sensitivity analysis excluding the lowest decile of the population. The mediation effect remained robust and statistically significant (Proportion = 7.5%, *P* < 0.001) (Supplementary table S3), confirming that the pathway persists across a broad physiological spectrum. Second, to rule out the potential confounding effect of genetic phenotypes such as UGT1A1 polymorphisms, we performed an additional sensitivity analysis by excluding participants with total bilirubin levels > 1.2 mg/dL. In this subgroup, the mediating role of bilirubin persisted, accounting for 12.5% of the association between obesity and LTL, which is consistent with the primary findings (13.2% in the total population) (Supplementary Table S4). Third, the mediation effect was consistently significant across multiple sensitivity models with incremental adjustment for potential confounders, including hepatic enzymes, systemic inflammation (CRP), and metabolic indicators (glycohemoglobin and HDL-C), demonstrating the robustness of our primary findings (Supplementary Table S5). Fourth, in the sensitivity analysis excluding individuals with abnormal liver enzyme levels (ALT or AST > 40 U/L), the mediating role of bilirubin remained robust. After full adjustment, bilirubin accounted for 10.7% of the obesity-LTL association (Supplementary Table S6, *P* < 0.001). This confirms that the observed mediation is independent of subclinical liver disease. Finally, using waist circumference as an alternative exposure yielded consistent results, with bilirubin explaining 12.2% of the association (Supplementary Table S7). These results collectively confirm the robustness of the identified Obesity-Bilirubin-LTL axis.

## Discussion

Obesity and LTL have been shown to be inversely related in numerous studies [4–6]. Oxidative stress, inflammation, and insulin resistance are all thought to play a role in the relationship between obesity and LTL attrition [12, 28, 30–32]. Obesity and oxidative stress are positively correlated [11, 12], and the level of the antioxidant bilirubin, which is inversely correlated with oxidative stress in vivo, is again negatively correlated with LTL shortening [18]. Thus, there is a complex association between obesity, LTL, and total bilirubin. However, no study has explored whether total bilirubin plays a mediating role in the association between obesity and LTL shortening and to what extent this mediating role might be. In this study, we quantified the mediating effect of total bilirubin on the BMI‒LTL association and discovered that the positive relationship between obesity and LTL shortening was partially mediated through reduced total bilirubin levels, accounting for 13.2% of the effect. These findings indicate that oxidative stress may be a mediating factor in the connection between obesity and LTL attrition.

The proportion of the total association mediated by bilirubin was modest (13.2%). This magnitude implies that the vast majority (approximately 87.5%) of the effect of obesity on telomere shortening likely operates through other non-bilirubin pathways, such as chronic inflammation, insulin resistance, or direct lipotoxicity. Acknowledging this partial mediation adds scientific rigor by preventing an overestimation of the role of a single antioxidant. However, this magnitude should be interpreted within the multifactorial nature of leukocyte LTL regulation, which is influenced by a complex interplay of genetic, lifestyle, and environmental factors [33]. In such contexts, a single circulating biomarker accounting for over one-tenth of the variance for a complex trait may represent a meaningful and non-negligible biological contribution, consistent with effect sizes observed for other identified mediators in epidemiological studies of complex phenotypes [34]. The effect’s stability across sensitivity analyses indicates it is not a statistical artifact but represents a robust and distinct biological pathway. For complex traits like LTL, which are influenced by numerous factors, identifying a specific, stable pathway that mediates even a moderate proportion of the total effect is clinically informative. While this finding does not imply immediate clinical utility for individual prediction, it provides etiologic insight by highlighting a specific, modifiable pathway (bilirubin-antioxidant capacity) that partially links adiposity to a biomarker of cellular aging. This supports the biological plausibility of the model and identifies a target for future research to test in longitudinal and interventional settings.

A previous study by Hao et al. (2021) using the same NHANES dataset emphasized a non-linear, inverted U-shaped relationship between total bilirubin and LTL [18]. Our findings provide a more refined perspective on the bilirubin-LTL axis by accounting for a wide array of metabolic and inflammatory variables, including CRP, HDL-C, HbA1c, and ALT. By adjusting for these factors, we isolated the independent antioxidant role of bilirubin and found that the relationship follows a threshold saturation pattern rather than a significant inverted U-shape. This suggests that the protective benefits of bilirubin reach a plateau once adequate physiological levels (approximately 0.582 mg/dL) are achieved. While the mediation proportion attenuated from 13.1% to 7.5% after excluding the lowest decile, the continued significance of the effect underscores that the obesity-induced depletion of antioxidant reserves is a generalized mechanism affecting telomere maintenance across the population.

Our findings remained remarkably robust after excluding individuals with elevated bilirubin (> 1.2 mg/dL), who are more likely to carry UGT1A1 variants. The high degree of consistency in mediation proportions (12.5% vs. 13.1%) suggests that the protective role of bilirubin against obesity-induced telomere attrition is an intrinsic metabolic mechanism prevalent across the general physiological range, rather than a genetic artifact. Our sensitivity analysis, which excluded participants with elevated liver enzymes, revealed a persistent mediation proportion of 10.7%. This finding is crucial as it clarifies that the association is not merely an artifact of obesity-induced liver dysfunction or NAFLD. Instead, it supports the hypothesis that bilirubin acts through its intrinsic antioxidant capacity to buffer against obesity-related telomere shortening.

The biological plausibility of bilirubin serving as a link between metabolic status and cellular aging is supported by its well-established role as a potent endogenous antioxidant. At a physiological level, bilirubin effectively scavenges peroxyl radicals and inhibits the activation of NADPH oxidase, thereby attenuating oxidative damage to lipids, proteins, and DNA [35, 36]. This cytoprotective function is thought to underlie the consistent inverse associations observed in epidemiological studies between moderately elevated serum bilirubin levels and a lower risk of oxidative stress-mediated diseases, such as cardiovascular disease, metabolic syndrome, and type 2 diabetes [37, 38]. Consequently, bilirubin is conceptualized not merely as a waste product but as an integral component of the body’s antioxidant defense system. Its potential to mitigate oxidative stress provides a mechanistic rationale for investigating its role in pathways related to aging, including the preservation of leukocyte telomere length.

A critical consideration in interpreting our findings is the potential bidirectionality between obesity and serum bilirubin. While we modeled bilirubin as a mediator—hypothesizing that obesity-induced oxidative stress consumes antioxidant resources—emerging evidence suggests that bilirubin also functions as a metabolic hormone. Recent studies have demonstrated that bilirubin can act as an agonist for PPAR-alpha, thereby promoting thermogenesis and protecting against adiposity [39]. Under this paradigm, low bilirubin levels could be a contributing cause of obesity rather than merely a consequence. If low bilirubin levels predate and contribute to the development of obesity, bilirubin could technically function as a confounder in the obesity-LTL relationship. Given the cross-sectional nature of the NHANES 1999–2002 dataset, we cannot definitively establish the temporal sequence of these associations. However, the stability of our mediation model—even after adjusting for metabolic confounders like glycohemoglobin—supports the biological plausibility of the Obesity-Bilirubin-LTL axis. Future research utilizing Mendelian Randomization or prospective cohorts is essential to clarify these complex, potentially reciprocal relationships and to confirm whether bilirubin serves as a causal mediator or a prerequisite for metabolic health.

Given the cross-sectional design of NHANES, we cannot establish the temporal sequence of changes, and thus cannot infer causality from the observed associations. Changes in LTL may have preceded changes in the other variables. Furthermore, the relationships between BMI, total bilirubin, and LTL may be bidirectional, particularly for the link between BMI and LTL [4–6]. Therefore, the directional pathways depicted in Fig. 2 are not derived from the present data but are based on biological mechanisms proposed in prior literature [4, 11, 12]. Future longitudinal studies are needed to assess the potential bidirectional influences between changes in obestiy and LTL over time.

The present study possesses several key strengths. First, the use of a large, nationally representative cohort from NHANES ensures high generalizability of our findings to the broader US population. Second, to our knowledge, this is the first investigation to specifically quantify the mediating role of total bilirubin in the association between obesity and LTL shortening using a robust causal mediation framework. Third, our findings demonstrated remarkable stability across diverse adiposity metrics, including both BMI and waist circumference, indicating that the identified pathway reflects a consistent biological relationship rather than an artifact of a specific metric. Most importantly, the robustness of the Obesity-Bilirubin-LTL axis was further validated through an extensive series of sensitivity analyses. This comprehensive methodological approach reinforces the biological plausibility of the antioxidant exhaustion hypothesis in the context of obesity-related cellular aging.

Despite the comprehensive nature of our analysis and the inclusion of extensive sensitivity models, several limitations should be acknowledged. First, we exclusively measured telomere length in leukocytes. It is uncertain whether our findings can be applied to other tissues. However, studies have reported strong associations between LTL and telomere length [40]. Second, we utilized a complete-case analysis, which resulted in the exclusion of individuals with missing data. However, our rigorous comparison between the final analytical sample and the excluded eligible population demonstrated that the two groups remained comparable across the majority of major variables, suggesting that selection bias is likely minimal. Third, as this is a cross-sectional study based on the NHANES dataset, we cannot definitively establish temporal precedence or causal relationships. Although our mediation model is grounded in the biological hypothesis of antioxidant consumption, we cannot entirely rule out bidirectionality. Finally, while we adjusted for a comprehensive set of metabolic, inflammatory, and hepatic markers—including glycohemoglobin, CRP, and liver enzymes—residual or unmeasured confounding remains a possibility. Factors such as specific genetic polymorphisms, or more granular lifestyle and dietary data were not fully accounted for and may influence the observed associations. Future longitudinal studies are warranted to further validate the causal direction and long-term dynamics of the obesity-bilirubin-LTL axis.

## Conclusions

In conclusion, our study identified a significant association between obesity and LTL shortening, with serum bilirubin serving as a potential statistical mediator. These findings provide a biological rationale for the clinical importance of weight management, suggesting that maintaining a healthy BMI may help preserve endogenous antioxidant capacity (such as bilirubin levels) and, consequently, mitigate oxidative stress-induced telomere attrition. While the cross-sectional nature of this study precludes definitive causal inference, our results highlight bilirubin as a plausible integrative biomarker of metabolic and oxidative health. Future longitudinal studies are essential to validate these relationships and further elucidate the causal mechanisms underlying the obesity-bilirubin-LTL axis.

## Supplementary Information

Below is the link to the electronic supplementary material.

Supplementary Material 1

## Data Availability

The datasets generated and analyzed during the current study are available in the National Health and Nutrition Examination Survey database (https://www.cdc.gov/nchs/nhanes/).

## Abbreviations

BMI: Body mass index
ALT: Alanine aminotransferase
AST: Aspartate aminotransferase
CRP: C-reaction Protein
HDL: High-density lipoprotein cholesterol
LTL: Leukocyte telomere length
