# Supplementary material for: The association between obesity and telomere shortening is mediated through total bilirubin
Source: Cardiovasc Diabetol Endocrinol Rep. 2026 Jul 10;12:39. doi: 10.1186/s40842-026-00307-2 (PMC13352791; doi:10.1186/s40842-026-00307-2)
Supplement: Supplementary file 1 — Supplementary Material 1 [file 40842_2026_307_MOESM1_ESM.docx]

### **Supplementary material**

**Supplementary Table S1 Comparison of characteristics between included and excluded eligible participants**

**Supplementary Table S2 Threshold effect analysis of total bilirubin concentration and leukocyte telomere length using piece-wise linear regression**

**Supplementary Table S3 Sensitivity analysis of the mediating effect of bilirubin on the association between obesity and LTL after excluding the lowest decile of bilirubin**

**Supplementary Table S4 Sensitivity analysis of the mediating effect of bilirubin excluding participants with suspected UGT1A1 genetic variants (total bilirubin > 1.2 mg/dL)**

**Supplementary Table S5 Mediation Proportion of Bilirubin Across Different Adjustment Models**

**Supplementary Table S6 Sensitivity analysis of the mediating effect of bilirubin on the association between obesity and LTL after excluding participants with elevated liver enzymes (ALT or AST > 40 U/L)**

**Supplementary Table S7 Sensitivity mediation analysis using waist circumference as an alternative exposure for obesity**

**Supplementary Figure S1 Restricted cubic spline plot for the association between serum bilirubin levels and leukocyte telomere length**

**Supplementary Table S1. Comparison of characteristics between included and excluded eligible participants**

| **Variables** | Included Sample | Excluded Sample | P-value |
| --- | --- | --- | --- |
| ^a^ N | 6717 | 1110 |  |
| Age, years (mean±sd) | 44.9±16.4 | 47.6± 18.2 | 0.001 |
| Sex, male (%) | 52.8 | 42.2 | <0.001 |
| Race/ethnicity (%) | | | 0.256 |
| Non-Hispanic white | 72.9 | 71.7 |  |
| Non-Hispanic black | 9.1 | 11.6 |  |
| Mexican American | 6.9 | 5.8 |  |
| Other Hispanic | 7.2 | 6.1 |  |
| Other | 4.0 | 4.8 |  |
| Cigarette smoking (%) | | | 0.336 |
| Never | 50.5 | 48.1 |  |
| Past | 25.0 | 25.0 |  |
| Current | 24.5 | 26.9 |  |
| Alcohol consumption, gm (mean±sd) | 11.2±36.4 | 14.6±39.6 | 0.246 |
| ^b^ Physical activity, MET-based rank (%) | | | <0.001 |
| Sedentary | 19.6 | 39.5 |  |
| Low | 28.0 | 22.4 |  |
| Moderate | 19.4 | 15.3 |  |
| High | 33.0 | 22.8 |  |
| BMI, kg/m^2^ (mean±sd) | 28.1±6.3 | 27.8±6.1 | 0.363 |
| Waist circumference, cm (mean ±sd) | 95.9 ±15.7 | 95.5±15.0 | 0.632 |
| LTL, base pairs (mean±sd) | 5834.3 ±665.5 | 5834.1±787.7 | 0.995 |
| Total bilirubin, mg/dL (mean ±sd) | 0.7 ±0.3 | 0.7 ±0.3 | 0.018 |
| ALT, U/L(mean±sd) | 26.3 ±34.0 | 27.9±28.7 | 0.132 |
| AST, U/L(mean±sd) | 24.5 ±17.5 | 26.8±26.2 | 0.019 |
| CRP, mg/dL (mean±sd) | 0.4 ±0.7 | 0.4 ±0.8 | 0.554 |
| HDL, mg/dL (mean±sd) | 50.9 ±15.3 | 51.3±16.4 | 0.421 |
| Glycohemoglobin, % (mean±sd) | 5.4±0.9 | 5.5±1.1 | 0.048 |

Note:

Mean ± SD (standard deviation) for continuous variables, P value was calculated by weighted linear regression model. Number (%) for categorical variables, P value was calculated by weighted chi-square test.

^a^ Unweighted number of observations in dataset.

^b^ Physical activity categories were based on the distribution of MET -minute levels for the present NHANES sample.

Abbreviations: BMI, body mass index; ALT, alanine aminotransferase; AST, aspartate aminotransferase;

CRP, C-reaction Protein; HDL, high-density lipoprotein cholesterol; LTL: Leukocyte telomere length

**Supplementary Table S2 Threshold effect analysis of total bilirubin concentration and leukocyte telomere length using piece-wise linear regression**

| leukocyte telomere length (β (95% CI)) adjusted∗ | | P value |
| --- | --- | --- |
| Total bilirubin, mg/dL |  |  |
| <0.582 | 437.072 (185.74,688.404) | 0.001 |
| ≥0.582 | 10.392 (-70.253,91.037) | 0.801 |

*adjusted for age, sex, race, cigarette smoking, alcohol consumption, physical activity, ALT, AST, CRP, HDL, and glycohemoglobin. Abbreviations as Table 1.

**Supplementary Table S3 Sensitivity analysis of the mediating effect of bilirubin on the association between obesity and LTL after excluding the lowest decile of bilirubin**

| Item | Estimate | Lower 95%CI | Upper 95%CI | P value |
| --- | --- | --- | --- | --- |
| Average Causal Mediation Effects | -0.274 | -0.509 | -0.041 | 0.02 |
| Average Direct Effects | -3.399 | -6.247 | -0.877 | 0.006 |
| Total Effect | -3.673 | -6.52 | -1.166 | 0.004 |
| Proportion of Mediated | 0.075 | 0.01 | 0.251 | 0.024 |

Adjusted for age, sex, race, cigarette smoking, alcohol consumption, physical activity, ALT, AST, CRP, HDL, and glycohemoglobin. Abbreviations as Table 1.

**Supplementary Table S4 Sensitivity analysis of the mediating effect of bilirubin excluding participants with suspected UGT1A1 genetic variants (total bilirubin > 1.2 mg/dL)**

| Item | Estimate | Lower 95%CI | Upper 95%CI | P value |
| --- | --- | --- | --- | --- |
| Average Causal Mediation Effects | -0.447 | -0.696 | -0.231 | < 0.001 |
| Average Direct Effects | -3.132 | -5.763 | -0.608 | 0.014 |
| Total Effect | -3.578 | -6.23 | -1.1 | 0.004 |
| Proportion of Mediated | 0.125 | 0.048 | 0.421 | 0.004 |

Adjusted for age, sex, race, cigarette smoking, alcohol consumption, physical activity, ALT, AST, CRP, HDL, and glycohemoglobin. Abbreviations as Table 1.

**Supplementary Table S5 Mediation Proportion of Bilirubin Across Different Adjustment Models**

| Model | Covariates Adjusted | Mediation Proportion (%) | P-value |
| --- | --- | --- | --- |
| Model 1 | age, sex, race, cigarette smoking, alcohol consumption, physical activity | 12.5% | < 0.001 |
| Model 2 | Model 1 + ALT + AST | 12.9% | < 0.001 |
| Model 3 | Model 2 + CRP | 13.1% | < 0.001 |
| Model 4 | Model 3 + HDL-C + glycohemoglobin | 13.2% | 0.002 |

Abbreviations as Table 1.

**Supplementary Table S6 Sensitivity analysis of the mediating effect of bilirubin on the association between obesity and LTL after excluding participants with elevated liver enzymes (ALT or AST > 40 U/L)**

| Item | Estimate | Lower 95%CI | Upper 95%CI | P value |
| --- | --- | --- | --- | --- |
| Average Causal Mediation Effects | -0.512 | -0.796 | -0.263 | < 0.001 |
| Average Direct Effects | -4.273 | -6.874 | -1.58 | 0.004 |
| Total Effect | -4.785 | -7.486 | -2.053 | 0.002 |
| Proportion of Mediated | 0.107 | 0.048 | 0.261 | 0.002 |

Adjusted for age, sex, race, cigarette smoking, alcohol consumption, physical activity, ALT, AST, CRP, HDL, and glycohemoglobin. Abbreviations as Table 1.

**Supplementary Table S7 Sensitivity mediation analysis using waist circumference as an alternative exposure for obesity**

| Item | Estimate | Lower 95%CI | Upper 95%CI | P value |
| --- | --- | --- | --- | --- |
| Average Causal Mediation Effects | -0.217 | -0.335 | -0.112 | < 0.001 |
| Average Direct Effects | -1.558 | -2.59 | -0.545 | 0.002 |
| Total Effect | -1.775 | -2.809 | -0.801 | < 0.001 |
| Proportion of Mediated | 0.122 | 0.055 | 0.305 | < 0.001 |

Adjusted for age, sex, race, cigarette smoking, alcohol consumption, physical activity, ALT, AST, CRP, HDL, and glycohemoglobin. Abbreviations as Table 1.


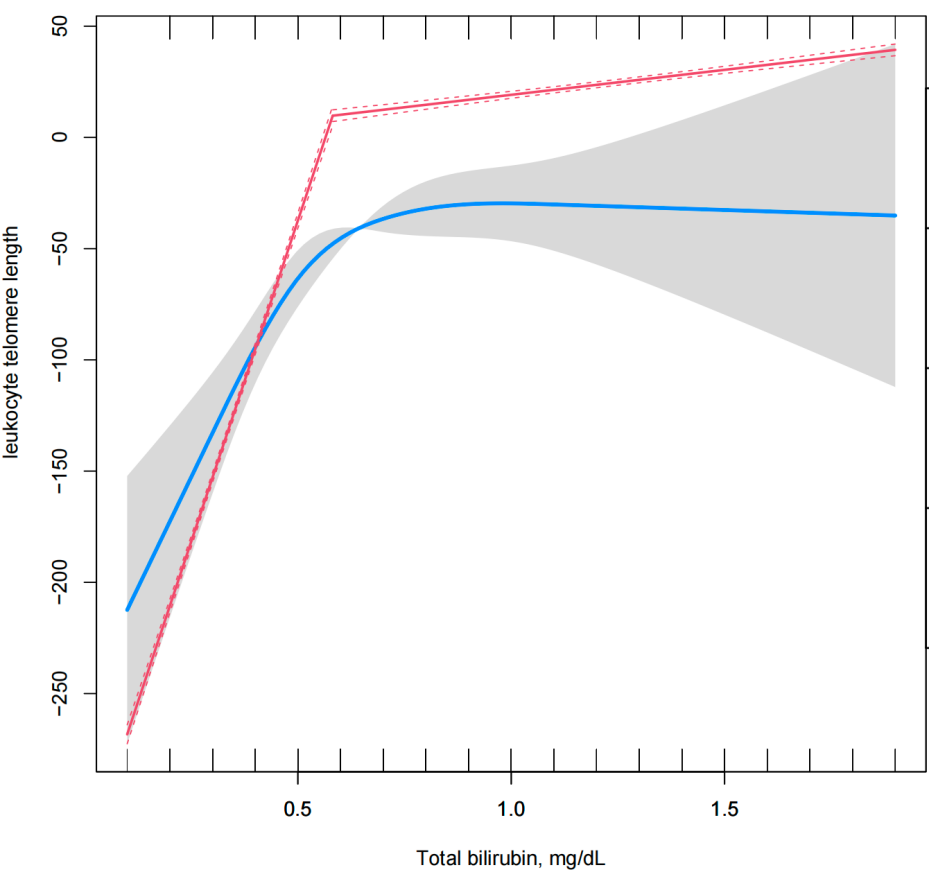


**Supplementary Figure S1 Restricted cubic spline plot for the association between serum bilirubin levels and leukocyte telomere length**

Adjust for age, sex, race, cigarette smoking, alcohol consumption, physical activity, ALT, AST, CRP, HDL, and glycohemoglobin.
